# CRP patterns and clinical associations in febrile patients undergoing autologous bone marrow transplant: experience from the sole transplant center in the West Bank, Palestine

**DOI:** 10.1007/s00277-026-06967-5

**Published:** 2026-03-26

**Authors:** Ibrahim ghoul, Maher Battat, Ahmad Khursani, Riad Amer, Mohamad Wild-Ali, Sawsan Muslm, Ramzi Shawahna, Mohammad Hayek

**Affiliations:** 1 Oncology and Hematology department, An-Najah National University Hospital, An-Najah National University, Nablus, Palestine; 2 Leukemia and Bone Marrow Transplantation Department, An-Najah National University Hospital, An-Najah National University, Nablus, Palestine; 3 Department of Physiology, Pharmacology and Toxicology, Faculty of Medicine and Health Sciences, An-Najah National University, Nablus, Palestine; 4 Faculty of Medicine and Health Sciences, Nursing and Midwifery Department, An- Najah National University, Nablus, Palestine

**Keywords:** Autologous Bone Marrow Transplantation (Auto-BMT), C-Reactive Protein (CRP), Febrile neutropenia, Inflammatory biomarkers, Hematopoietic stem cell transplantation, White blood cell engraftment, Fever patterns, Conditioning regimens, Oncology outcomes and post-transplant monitoring

## Abstract

Autologous bone marrow transplantation (auto-BMT) is frequently complicated by febrile episodes and systemic inflammation, particularly during the neutropenic phase, making inflammatory markers such as C-reactive protein (CRP) valuable for monitoring recovery and guiding clinical decisions. In this descriptive, retrospective study of 244 febrile auto-BMT patients in the West Bank, data on demographics, CRP levels (days 1–14), fever onset and duration, and white blood cell (WBC) engraftment were analyzed using non-parametric tests and Spearman correlation. Fever typically began around day 6.7, lasted 6.5 days, and was accompanied by a CRP peak on day 8 (mean 104.8 mg/L) before gradually declining, while WBC engraftment occurred on day 12.96 (SD ± 5.29). CRP levels significantly differed by cancer type and conditioning regimen (*p* < .001), but not by gender, age, or comorbidities, and were weakly correlated with fever duration and negatively with fever onset. WBC engraftment day showed moderate correlations with fever duration and end. These findings suggest that CRP and fever patterns follow a predictable inflammatory course peaking around day 8, with engraftment timing significantly affecting fever duration, underscoring CRP’s potential as a supportive biomarker for post-transplant monitoring.

## Background

This study draws on clinical data from the only center offering autologous bone marrow transplantation (auto-BMT) in the West Bank, a region facing chronic resource limitations and frequent disruptions in medical supply chains. The data were collected to understand the dynamics of post-transplant fever and inflammation in febrile auto-BMT patients and to generate locally relevant evidence that supports clinical decision-making in a low-resource, high-need setting. Auto-BMT is often complicated by infectious and inflammatory events due to the profound neutropenia induced by conditioning regimens. Bacterial infections, particularly bacteremia caused by viridans streptococci, occur frequently, with septicemia reported in up to 8% of patients alongside skin and urinary tract infections [1–5]. Fungal infections are usually mucocutaneous, with invasive forms occurring in about 2% of cases [1–5]. Viral infections and pulmonary complications both infectious and non-infectious also contribute to morbidity and mortality [6–8]. Inflammatory syndromes such as SIRS are common during marrow recovery and are linked to endothelial injury and respiratory failure [3, 9].

Routine surveillance includes monitoring for fever, inflammatory markers (e.g., CRP, cytokines), blood cultures, and imaging [3, 6, 9]. Elevated CRP is associated with increased infection risk, prolonged neutropenia, and non-relapse mortality [10–12]. Though nonspecific, CRP is often used to guide treatment decisions and may be more reliable when used with biomarkers like procalcitonin or IL-6 [13, 14]. Despite growing interest in CRP’s utility, existing studies are often limited by small sample sizes, retrospective designs, and single-center data from high-income countries, limiting their generalizability to low-resource settings [10, 11, 14]. Therefore, evidence from the West Bank can provide critical insights into inflammatory trajectories and outcomes in underrepresented populations.

Although CRP is widely used in hematopoietic stem cell transplantation, existing studies have primarily focused on allogeneic transplantation or on small, heterogeneous cohorts from high-income countries. Data specifically describing the temporal relationship between CRP, fever, and engraftment in autologous transplantation remain limited, and virtually absent from low-resource settings. Moreover, prior studies have largely examined CRP as a predictor of complications or mortality, rather than describing its daily trajectory in relation to fever onset and resolution. This lack of descriptive evidence represents an important gap, particularly for centers where access to advanced biomarkers is restricted and CRP remains the most readily available inflammatory marker. The present study was therefore undertaken to characterize CRP dynamics alongside fever and WBC recovery in auto-HCT patients and to evaluate whether these routinely collected parameters demonstrate consistent and clinically interpretable patterns.

The objective of this study is to describe and analyze patterns of CRP elevation, fever duration, and their association with white blood cell (WBC) engraftment in febrile auto-BMT patients. By examining these relationships in a real-world, resource-limited environment, this work contributes valuable evidence for tailoring supportive care strategies and refining risk assessment in both local and global transplant settings.

## Methodology

This retrospective, population-based, cross-sectional analytical study was conducted to assess fever onset, duration, and C-reactive protein (CRP) patterns in febrile patients undergoing aauto-BMT) at An-Najah National University Hospital the sole center offering auto-BMT services in the West Bank. This allowed the inclusion of the entire regional population of febrile transplant patients from 2014 to 2024.

### Study population and setting

All adult auto-BMT recipients who developed fever during the neutropenic phase and had complete CRP data from Days + 1 to + 14 post-transplant were included. Of 300 patients, 244 met inclusion criteria, contributing 3,416 CRP values. Eighteen (6%) patients had no fever, and 41 febrile patients were excluded due to missing CRP data.

### Data collection

Data were extracted retrospectively from electronic and paper-based records using a structured form. Variables included demographics, cancer type, comorbidities, conditioning regimens, WBC engraftment day, fever onset/end, and daily CRP levels. Data on fever and white blood cell counts were collected from the onset of neutropenia until hospital discharge. Fever data were collected daily from the onset of neutropenia until hospital discharge. No statistical imputation was performed for missing values; analyses were conducted using available data only. For the purposes of this analysis, CRP values were restricted to Days + 1 through + 14 post-transplant, reflecting the period of greatest inflammatory activity and the timeframe during which CRP was routinely ordered for all patients. Length of hospital stay was not used as an analytical variable but was inherently linked to the period of clinical observation.

### Operational definitions

Fever was defined as ≥ 38.3 °C once or ≥ 38.0 °C for over an hour. WBC engraftment was the first of three days with neutrophils ≥ 500/µL. Peak CRP day was the highest CRP value between Days + 1 and + 14 [15, 16].

### Data analysis

SPSS version 21 was used. Data normality was tested using Shapiro-Wilk. Descriptive and inferential statistics were applied based on variable type and distribution. *p* < .05 was considered significant. No deaths occurred during the clinical observation period; therefore, mortality-related analyses were not performed.

## Results

### Participant demographics and clinical characteristics

**Fig. 1:**
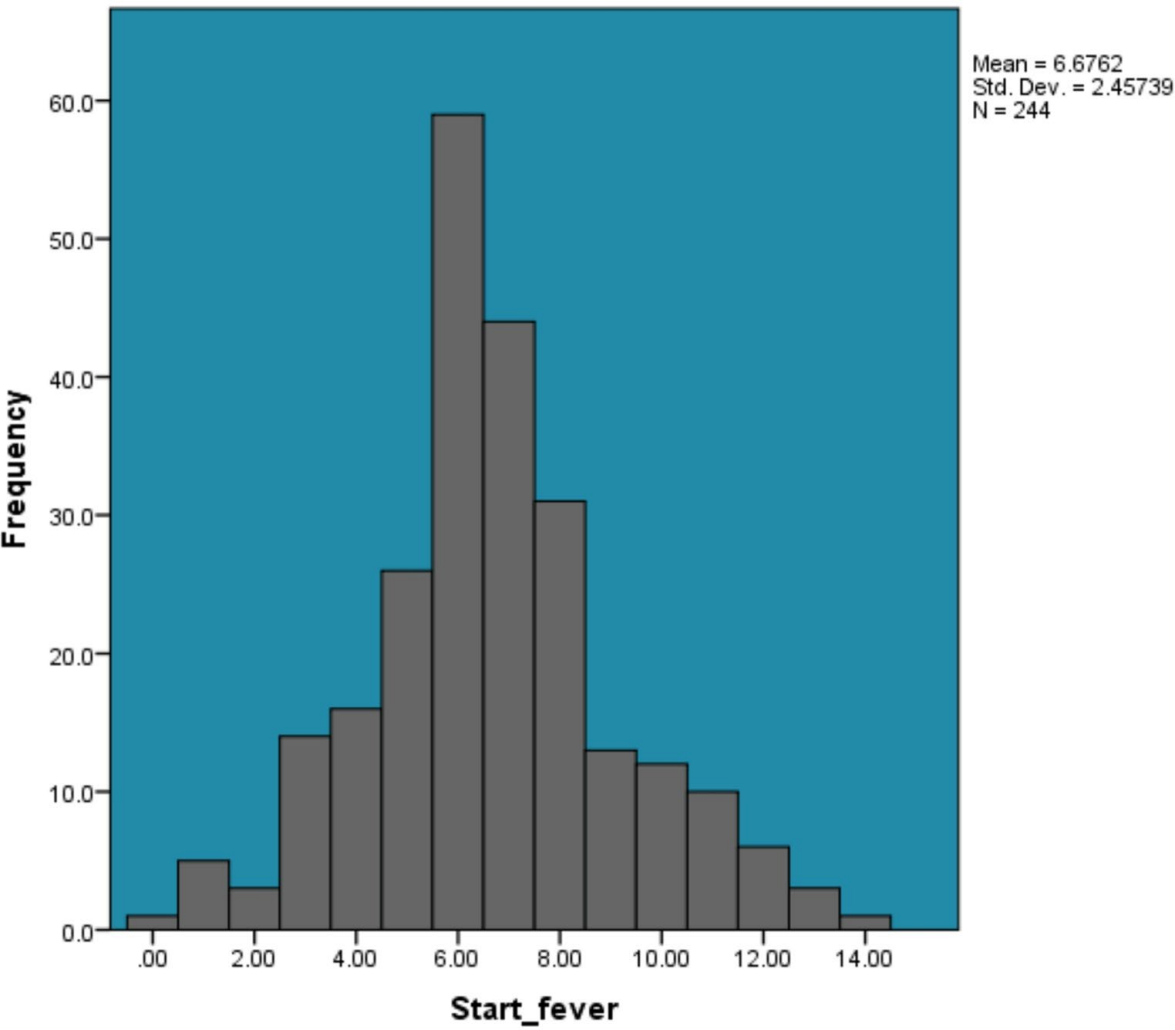
Percentage distribution of initial fever onset days following autologous bone marrow transplantation (*N* = 244)

A total of 3,416 CRP values were analyzed from 244 auto-BMT patients (54.5% male; mean age 45.5 ± 14.0 years). Multiple myeloma was the most common cancer (53.7%), followed by Hodgkin lymphoma (32.4%) and non-Hodgkin lymphoma (13.9%). Hypertension affected 27% and diabetes 16.4% of patients. Conditioning regimens included Melphalan (53.3%) and LEAM (46.7%). The period of clinical observation extended from transplantation until hospital discharge, with a median duration of clinical observation of 16 days (IQR 14–19). In Fig. 1, fever began on average at day 6.68 (± 2.46), ending at day 12.14 (± 6.00), with durations ranging from 1 to 66 days (mean 6.46 ± 6.04). These results reflect a varied fever pattern, with significant differences in onset and resolution among patients. No deaths occurred during the clinical observation period; therefore, mortality-related analyses were not performed.

### CRP trends and peak day distribution

**Fig. 2:**
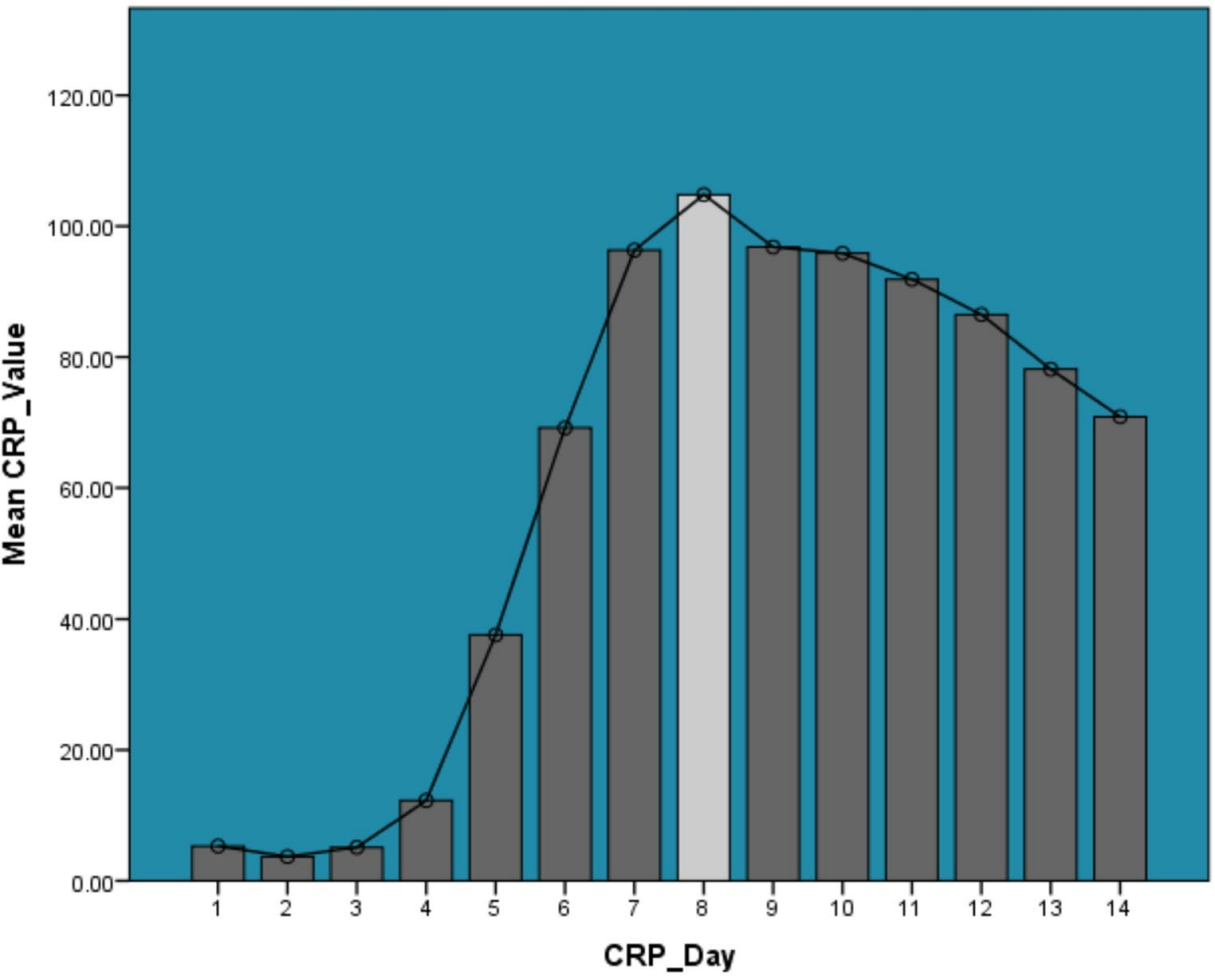
Mean daily c-reactive protein (CRP) levels during the First 14 days post-autologous bone marrow transplantation

CRP levels in 244 auto-BMT patients rose sharply from day 5, peaking on day 8 with a mean of 104.82 mg/L (SD 73.71), then gradually declined but remained elevated through day 14 (mean 70.87 mg/L, SD 73.70) (Fig. 2). This reflects the typical inflammatory peak post-transplant.

### White blood cell engraftment timing

Among 244 patients, mean WBC engraftment occurred at 12.96 days (SD ± 5.29), ranging from 1 to 39 days post-transplant. Most engrafted by the second week, but some experienced delayed recovery, highlighting the need for monitoring due to its impact on infection risk, neutropenia duration, and fever length.

### Association between CRP, fever duration, and clinical variables

Using the Shapiro-Wilk test, all continuous variables (CRP, age, WBC engraftment day, fever onset, end, and duration) had p-values < 0.001, indicating non-normal distributions; thus, non-parametric tests were used. Table 1 shows no significant differences in CRP by gender (*p* = .991), hypertension (*p* = .928), or diabetes (*p* = .090) Although a higher proportion of patients with diabetes experienced the outcome of interest, this association did not reach statistical significance (*p* = .09). The small number of diabetic patients in the cohort may have limited the ability to detect a statistically significant difference. Significant differences were found by conditioning regimen and cancer type (both *p* < .001), suggesting variability in inflammatory responses. Spearman correlation showed no significant relationship between CRP and age or WBC engraftment day. However, CRP showed weak but significant correlations with fever duration (rₛ = 0.095, *p* < .001), fever end (rₛ = 0.035, *p* = .039), and a negative correlation with fever onset (rₛ = -0.096, *p* < .001). Age was weakly associated with later fever onset (rₛ = 0.183) and engraftment (rₛ = 0.105). WBC engraftment day was significantly correlated with fever duration (rₛ = 0.157, *p* < .001) and fever end (rₛ = 0.318, *p* < .001). The strongest correlation observed was between fever end and duration (rₛ = 0.751, *p* < .001).

**Table 1:** Non-parametric group comparisons (Mann–Whitney U and Kruskal–Wallis) between CRP, and clinical variables

Variable (Grouping)	Test Used	Test Statistic (U / χ²)	Z-Value	df	*p*-value	Interpretation
Gender	Mann–Whitney U	1,446,464	-0.011	—	0.991	No significant difference in CRP between males and females
Hypertension (HTN)	Mann–Whitney U	1,142,549	-0.090	—	0.928	No significant difference in CRP between HTN and non-HTN patients
Diabetes Mellitus (DM)	Mann–Whitney U	763,458	-1.697	—	0.090	No significant difference, but close to borderline (*p* = .090)
Conditioning Regimen	Mann–Whitney U	1,338,791	-3.949	—	0.000	Significant difference in CRP by regimen
Cancer Type (3 groups)	Kruskal–Wallis	χ² = 26.508	—	2	0.000	Significant difference in CRP between cancer types

## Discussion

Our study of 244 patients undergoing autologous bone marrow transplantation (auto-BMT) reveals a clinical profile that aligns with existing literature. The nearly equal gender distribution and a mean age of 45.5 years reflect a typical middle-aged adult population commonly seen in auto-BMT cohorts [17]. The most frequent diagnosis was multiple myeloma (53.7%), followed by Hodgkin lymphoma (32.4%) and non-Hodgkin lymphoma (13.9%), consistent with current practices where auto-BMT is standard for relapsed or refractory cases [17, 18]. Comorbid conditions were common, with hypertension present in 27% and diabetes mellitus in 16.4%, reinforcing the importance of chronic disease management during transplant care [17]. Melphalan and LEAM were the main conditioning regimens, aligning with standard protocols for myeloma and lymphoma, respectively [17].

Fever onset typically occurred around day 6.68 (± 2.46) post-transplant and ended by day 12.14 (± 6.00), with a mean duration of 6.46 days, ranging from 1 to 66 days. This timeline reflects the neutropenic phase and the start of engraftment, when immune suppression is highest and infection risk peaks [2, 4, 19]. The variability in fever duration supports previous studies noting that many fevers in this setting are not linked to documented infections and may be due to sterile inflammation or engraftment syndrome [2, 20]. Persistent fever in some patients may relate to prolonged neutropenia or post-conditioning inflammatory complications, emphasizing the need for individualized monitoring. CRP patterns showed a clear trend: values were low in the first few days, began rising on day 5, peaked at day 8 (mean 104.82 mg/L), and remained elevated through day 14 (mean 70.87 mg/L). This trajectory matches the expected acute-phase inflammatory response following transplantation and conditioning regimens [9, 21]. The CRP peak corresponds with findings in allogeneic transplant studies showing rises in inflammatory markers between days 5–11, signaling systemic inflammation or infectious risk [21]. Persistently high CRP after day 8 suggests ongoing inflammation or tissue repair and may serve as an early indicator of post-transplant complications [22–24]. WBC engraftment occurred at a mean of 12.96 days (± 5.29), ranging from 1 to 39 days. This is consistent with established benchmarks for auto-BMT, where neutrophil recovery typically begins between days 10–16 [25–27]. The wide range highlights variability influenced by factors such as stem cell dose, patient characteristics, and prior therapies [28]. Timely engraftment reduces the risk of infection and fever, while delays may require increased supportive care [25, 26]. CRP values did not differ significantly by gender (*p* = .991), hypertension (*p* = .928), or diabetes (*p* = .090). This suggests that CRP levels are more influenced by transplant-specific factors than baseline patient demographics [9]. Significant differences in CRP were observed by conditioning regimen and cancer type (*p* < .001), indicating that disease characteristics and treatment intensity shape the inflammatory response [9]. No correlation was found between CRP and age or engraftment day. However, CRP correlated weakly but significantly with fever duration (rₛ = 0.095, *p* < .001), fever end (rₛ = 0.035, *p* = .039), and inversely with fever onset (rₛ = -0.096, *p* < .001), showing modest alignment between CRP elevations and fever dynamics [9, 13]. WBC engraftment correlated with both fever duration (rₛ = 0.157, *p* < .001) and fever end day (rₛ = 0.318, *p* < .001), underlining its impact on fever resolution. The strongest correlation overall was between fever duration and fever end day (rₛ = 0.751, *p* < .001), as expected due to their related definitions. These findings confirm that while demographic factors have limited effect on CRP levels, treatment-related variables and engraftment kinetics significantly influence the inflammatory course [9, 11]. The association between CRP levels and both cancer type and conditioning regimen suggests that treatment-related tissue injury and disease-specific inflammatory responses contribute meaningfully to post-transplant inflammation. More intensive regimens and certain malignancies may induce greater mucosal and endothelial damage, thereby amplifying CRP responses independent of infection. These findings underscore the need to interpret CRP values within the context of transplant characteristics rather than as universal thresholds applicable to all auto-HCT patients.

The findings of this study do not support a change in antimicrobial practice based solely on CRP values; however, they provide important contextual information for interpreting CRP trends during the neutropenic phase. The observation that fever onset generally precedes peak CRP suggests that CRP primarily reflects the downstream inflammatory response rather than serving as an early trigger for intervention. Transplant-specific factors may also influence inflammatory responses following auto-BMT. In this cohort, multiple myeloma patients predominantly received high-dose melphalan conditioning, whereas lymphoma patients primarily received LEAM. Both regimens are considered intensive and can induce mucosal injury and systemic inflammatory responses, which may contribute to elevations in CRP levels independent of infection. Therefore, variations in CRP patterns observed in this study may partially reflect differences in conditioning-related tissue injury rather than infection alone. The temporal relationship between fever onset and CRP elevation observed in this study suggests that CRP may not serve as an early anticipatory marker of infection in auto-BMT patients. Instead, CRP elevations tended to follow or coincide with febrile episodes and frequently remained elevated even as patients clinically improved and were discharged. These findings suggest that CRP should be interpreted cautiously and in conjunction with clinical assessment rather than used in isolation for diagnostic decision-making. Thus, CRP should be interpreted as a supportive marker rather than a diagnostic or decision-making tool in isolation. In this cohort, rising or plateauing CRP levels were not linked to specific outcomes or predefined clinical actions, and therefore cannot be used to justify antibiotic escalation or de-escalation independently. Instead, daily CRP monitoring may help clinicians recognize expected inflammatory trajectories and identify deviations from typical patterns that warrant closer clinical assessment. Visual inspection of CRP and fever trajectories demonstrates that fever onset typically occurred prior to peak CRP levels. This temporal pattern indicates that CRP elevation reflects a subsequent inflammatory response rather than an early marker of febrile events. Accordingly, CRP trends appear to mirror the course of systemic inflammation rather than anticipate clinical deterioration.

### Limitations

This study is limited by its single-center, retrospective design, which may restrict generalizability. Potential confounders and documentation gaps are inherent limitations. Other biomarkers like procalcitonin, IL-6, or ferritin were not assessed, which could improve diagnostic accuracy. Additionally, analysis was restricted to the first 14 days post-transplant, potentially missing late-onset complications.

Another limitation is that CRP measurements were analyzed only through Day + 14 post-transplant. Although CRP levels remained elevated at this time point in many patients, additional measurements beyond Day + 14 were not consistently available. Furthermore, many patients were discharged while CRP levels were still above the normal range, suggesting that CRP may lag behind clinical recovery and therefore may have limited utility as a standalone marker for monitoring resolution of inflammation in this population. Additionally, the relatively small number of patients with diabetes limited the statistical power to evaluate its potential association with CRP trends or clinical outcomes.

Future studies should adopt multicenter, prospective designs and include broader biomarker panels to differentiate between infectious and sterile inflammation. Extending the observation period beyond day 14 may capture late complications. Developing a composite risk score incorporating CRP trends, engraftment timing, and clinical variables could enhance early risk identification, especially in resource-limited settings like the West Bank.

## Conclusion

This study provides a comprehensive overview of fever patterns, CRP dynamics, and WBC engraftment in patients undergoing auto-BMT in the West Bank. The findings reveal that fever commonly begins around day 7 post-transplant and lasts on average for 6.5 days, while CRP levels follow a predictable inflammatory trend, peaking on day 8. WBC engraftment typically occurs around day 13, though with wide individual variability. Importantly, the inflammatory response as measured by CRP varied significantly by cancer type and conditioning regimen, but not by gender, age, or comorbidities such as hypertension or diabetes. Weak yet statistically significant correlations were found between CRP levels and fever duration, fever onset, and fever end, suggesting that CRP is a modest but useful marker for tracking febrile inflammation during neutropenia. Furthermore, WBC engraftment day was positively correlated with fever end and fever duration, underlining its role in resolving infection-related complications. These results highlight the potential of daily CRP monitoring to complement clinical judgment in managing febrile episodes and predicting recovery during the post-transplant period. The patterns observed serve as a reference for expected clinical trajectories in auto-BMT patients and may guide early interventions in cases of delayed engraftment or prolonged inflammation.

## Data Availability

The data that support the findings of this study are available from the corresponding author upon a reasonable request.

## Abbreviations

CRP: C-reactive protein
WBC: White blood cell
BMT: Bone marrow transplantation

## Declarations

### Clinical trial registration

This research did not involve a clinical trial; no clinical trial registration is applicable.
